# Induction of a Peptide with Activity against a Broad Spectrum of Pathogens in the *Aedes aegypti* Salivary Gland, following Infection with Dengue Virus

**DOI:** 10.1371/journal.ppat.1001252

**Published:** 2011-01-13

**Authors:** Natthanej Luplertlop, Pornapat Surasombatpattana, Sirilaksana Patramool, Emilie Dumas, Ladawan Wasinpiyamongkol, Laure Saune, Rodolphe Hamel, Eric Bernard, Denis Sereno, Frédéric Thomas, David Piquemal, Hans Yssel, Laurence Briant, Dorothée Missé

**Affiliations:** 1 Department of Tropical Hygiene, Faculty of Tropical Medicine, Mahidol University, Bangkok, Thailand; 2 Laboratoire Maladies Infectieuses et Vecteurs: Ecologie, Génétique, Evolution, Contrôle, UMR 224 CNRS/IRD/UM1, Montpellier, France; 3 Centre d'études d'agents Pathogènes et Biotechnologies pour la Santé (CPBS), CNRS UMR 5236-UM1-UM2, Montpellier, France; 4 Institut de Recherche en Biologie Végétale (IRV), Université de Montréal, Montreal, Quebec, Canada; 5 University of Montpellier II, Montpellier, France; 6 Inserm, U844, Montpellier, France; Washington University School of Medicine, United States of America

## Abstract

The ultimate stage of the transmission of Dengue Virus (DENV) to man is strongly dependent on crosstalk between the virus and the immune system of its vector *Aedes aegypti (Ae. aegypti)*. Infection of the mosquito's salivary glands by DENV is the final step prior to viral transmission. Therefore, in the present study, we have determined the modulatory effects of DENV infection on the immune response in this organ by carrying out a functional genomic analysis of uninfected salivary glands and salivary glands of female *Ae. aegypti* mosquitoes infected with DENV. We have shown that DENV infection of salivary glands strongly up-regulates the expression of genes that encode proteins involved in the vector's innate immune response, including the immune deficiency (IMD) and Toll signalling pathways, and that it induces the expression of the gene encoding a putative anti-bacterial, cecropin-like, peptide (AAEL000598). Both the chemically synthesized non-cleaved, signal peptide-containing gene product of AAEL000598, and the cleaved, mature form, were found to exert, in addition to antibacterial activity, anti-DENV and anti-Chikungunya viral activity. However, in contrast to the mature form, the immature cecropin peptide was far more effective against Chikungunya virus (CHIKV) and, furthermore, had strong anti-parasite activity as shown by its ability to kill *Leishmania* spp. Results from circular dichroism analysis showed that the immature form more readily adopts a helical conformation which would help it to cause membrane permeabilization, thus permitting its transfer across hydrophobic cell surfaces, which may explain the difference in the anti-pathogenic activity between the two forms. The present study underscores not only the importance of DENV-induced cecropin in the innate immune response of *Ae. aegypti*, but also emphasizes the broad-spectrum anti-pathogenic activity of the immature, signal peptide-containing form of this peptide.

## Author Summary

Dengue viruses (DENV) are generally maintained in a cycle which requires horizontal transmission *via* their arthropod vector, *Ae. aegypti*, to the vertebrate host. One important consequence of this process is the interference of the virus with the immune systems of both the mosquito and its host. While infection of humans causes disease, the presence of DENV in mosquitoes gives rise to life-long and persistent infection with active viral replication in the salivary glands. In the present study, we have evaluated the mosquito's immune response following DENV infection by analyzing the gene expression profile of infected and uninfected salivary glands. The results show that DENV infection activates signaling pathways and induces the expression of gene products that are involved in the innate immune response to DENV infection, and in particular a putative antibacterial cecropin-like peptide. The immature and mature forms of this peptide were found to be active against a variety of pathogens including DENV and Chikungunya viruses, as well as the *Leishmania* parasite. This study is the first to establish a comparative analysis of uninfected salivary glands and salivary glands of female *Ae. aegypti* mosquitoes infected with DENV. We demonstrate that certain DENV-induced peptides possess broad-spectrum anti-pathogenic activity and may have therapeutic potential in the treatment of human infectious disease.

## Introduction

The recent emergence of dengue constitutes a serious health threat and today, the disease is considered one of the most serious arthropod-borne human viral diseases in terms of both morbidity and mortality [1], with approximately 50–100 million new infections per annum, including 200,000–500,000 cases of potentially life-threatening dengue hemorrhagic fever or dengue shock syndrome [2], [3] which is the leading cause of infant mortality in several Asian countries. The National Institute of Allergy and Infectious Diseases has classified DENV as a category A biothreat pathogen. There is currently no licensed vaccine or drug treatment against this pathogen and, at present, the only method of preventing transmission is by controlling its vector, the *Ae. aegypti* and *Aedes albopictus (Ae. albopictus)* mosquitoes. Dengue viruses circulate in nature as four distinct serological types (DENV-1 to -4) which are ingested by the vector when it feeds on an infected host. The virus then crosses the gut epithelium into the hemolymph to reach the salivary glands *via* various routes. Once in the insect's saliva, it is inoculated into a vertebrate host to recommence its replicative cycle [4].

An important consequence of this transmission process is the interference of DENV with both the invertebrate and human immune systems. While infection of vertebrates causes severe disease, the presence of DENV in mosquitoes is non-pathogenic and results in life-long, persistent infection. This difference may reflect the capacity of insects to mount a highly effective innate immune response of both cellular and humoral nature to control invading microbes [5], [6].

The immune pathways and effectors activated in response to pathogens in *Ae. aegypti* still remain elusive. However, recent studies have shown that oral infection with DENV elicits dsRNA and production of DENV-specific siRNAs in the midgut [7], supporting a major role for the RNAi pathway in the control of DENV replication in *Ae. aegypti*. In addition, the Toll and the Janus kinase/signal transduction and activators of transcription (JAK-STAT) pathways have also been reported to play an important role in controlling DENV replication in the midgut [8], [9]. Finally, the recent sequencing and annotation of the *Ae. aegypti* genome is of importance to identify new effectors in the immune system of this vector [10].

However, although the salivary gland of *Ae. aegypti* is the final organ required to be infected prior to the transmission of DENV to humans, the complex relationship between this organ and the virus remains unknown. Antimicrobial peptides (AMP), lysozyme, and pathogen pattern recognition receptors are commonly found in the salivary gland of many hematophagous arthropods, pointing to the importance of this organ in the vector's immune defense mechanism [11], [12]. For example, a recently discovered tick protective antigen, subolesin, reportedly is active against pathogens in the salivary gland *via* NF-kB-dependent and independent signal transduction pathways that regulate innate immune responses [13]. Moreover, several defense response genes have been found in the salivary gland EST clusters of biting midge *Culicoides sonorensi* [14]. It is of note that a comparative transcriptome analysis of plasmodium-infected *Anopheles gambiae* salivary glands also revealed the induction of several innate immune response genes, encoding proteins such as prophenoloxidases and AMP [15].

In order to decipher the complex relationship between DENV and the innate immune system of *Ae. aegypti*, we have investigated whether the expression of some acute-phase proteins is modulated in salivary glands following infection of the vector by DENV. To this aim, using the Digital Gene Expression (DGE) analysis combined with a deep sequencing approach, we have carried out a comparative genomic analysis of uninfected salivary glands and salivary glands of female *Ae. aegypti* mosquitoes infected with DENV (referred to in the present study as uninfected and DENV-infected salivary glands). Among the up-regulated genes, we have identified a putative antibacterial peptide, belonging to the cecropin family, indicating that the IMD pathway is involved in the mosquito's defense against DENV in salivary glands. We further demonstrate that the immature, signal peptide-containing form of this peptide displays wide anti-infectious properties and is active against DENV, Chikungunya virus, as well as the protozoan parasite *Leishmania*.

## Materials and Methods

### Ethics statement

Venous blood from anonymous healthy human volunteers was obtained from the blood bank (Etablissement Français du Sang) in accordance with its guidelines, published in the French Journal Officiel, with informed written consent from each volunteer. The study was conducted according to the guidelines of the Institutional Review Board of the Institut de Recherche pour le Développement and was approved by the Institutional Review Board of the Institut de Recherche pour le Développement.

### Cells and viruses

*Ae. albopictus* C6/36 cells, used for the propagation of the four dengue serotypes DENV-1 (Hawaii strain [16]), DENV-2 (16681 strain [16]), DENV-3 (H87 strain [16]) and DENV-4 (814669 strain [17]) were grown in M199 medium (Invitrogen, France), supplemented with 10% fetal calf serum (FCS, Lonza, Switzerland) at 28°C. All DENV serotypes were passed three times in C6/36 cells. HEK-293T (Human Embryonic Kidney 293) and LLC-MK2 (Monkey kidney epithelial) cells were grown in DMEM, 10% FCS, at 37°C.

### Mosquito infection and sample preparation

The *Ae. aegypti* mosquito strain used in our study was a Liverpool strain originating from West Africa which has been maintained at the Liverpool School of Tropical Medicine since 1936 [18]. Mosquitoes were reared and maintained at 26°C±0.5°C with 75–80% relative humidity and a 12∶12 h (light∶dark) photoperiod. Infectious blood meals were offered 3 days post-emergence to adult, female, *Ae. aegypti* mosquitoes using a silicone membrane feeder system [19]. Human blood was combined (1∶1) with the culture supernatant of C6/36 cells infected with DENV-2 16681 to provide a titrated blood meal of 5.10^6^ plaque forming units (PFU)/ml. For the control group of mosquitoes, human blood was mixed in the same proportion with culture supernatant of uninfected C6/36 cells. Mosquitoes that failed to feed were discarded. At different time-points after the blood meal, salivary glands were carefully dissected in Phosphate Buffered Saline (PBS) and washed 3 times with PBS to prevent contamination with the fat body [11]. Each pair of salivary gland was frozen separately at −80°C in 20 µL acid guanidium thiocyanate-phenol-chloroform solution (RNable Eurobio, France). Before use of salivary glands for further experiments, the RNA of carcasses corresponding to the group of mosquitoes fed with infectious blood meal was extracted individually and tested for the presence of DENV by semi-quantitative PCR. Glands of mosquitoes containing DENV were then pooled (40 pairs of salivary for each time point) and lysed with 500 µL of RNable. RNA was precipitated following the addition of isopropanol, collected by centrifugation at 12000×g for 15 minutes and washed once with 75% ethanol. After a brief air-drying, the pellet was dissolved in RNase/DNase free water.

### RT-qPCR procedures for DGE analysis

RT-PCR was carried out on 2 µg of salivary gland RNA using the QIAGEN OneStep RT-PCR kit and 15 pmol of specific primers: DF: 5′ TCA-ATA-TGC-TGA-AAC-GCG-CGA-GAA-GAA-ACC-G3′; DR: TTG-CAC-CAA-CAG-TCA-ATG-TCT-TCA-GGT-TC3′. The PCR steps were: 50°C for 30 min, 95°C for 15 min, 39 cycles (94°C for 1 min, 55°C for 30 sec, 72°C for 1 min), 72°C for 10 min. The quality of the total RNA was checked by capillary electrophoresis analysis using an Agilent BioAnalyser 2100 (Agilent, Palo Alto, CA, USA) and quantities were measured using a NanoDrop ND-1000 spectrophotometer (Thermo Scientific, Les Ulis, France).

### Sequence tag preparation

Sequence tags were prepared with the Illumina Digital Gene Expression Tag Profiling Kit following the manufacturer's protocol (version 2.1B). One microgram of total RNA was incubated with oligo-dT beads to capture the polyadenlyated RNA fraction. First- and second-strand cDNA synthesis were performed while the RNA was bound to the beads. Still on the beads, samples were digested with NlaIII to retain a cDNA fragment from the most 3′ CATG to the poly (A)-tail. Subsequently, the GEX adapter 1 was ligated to the free 5′ end of the RNA, and the sample was digested with MmeI which cuts 17 bp downstream of the CATG site. At this point, the fragments detach from the beads. After dephosphorylation and phenol extraction, the GEX adapter 2 was ligated to the 3′ end of the tag. To enrich for the desired fragments, 15 cycles of PCR amplification with the Phusion polymerase (Finnzymes Espoo, Finland) were performed with primers complementary to the adapter sequences. The resulting 85 bp fragments were purified by excision from a 6% polyacrylamide TBE gel. The DNA was eluted with 1× NEBuffer 2 by gentle rotation for 2 h at room temperature. Gel debris was removed using a Spin-X Cellulose Acetate Filter (2 ml, 0.45 µm) and the DNA was precipitated by adding 10 µl of 3 M sodium acetate (pH 5.2) and 325 µl of ethanol (−20°C), followed by centrifugation at 14,000 rpm for 20 min. After washing of the pellet with 70% ethanol, the DNA was resuspended in 10 µl of 10 mM Tris–HCl, pH 8.5 and quantified using a Nanodrop 1000 spectrophotometer.

### Sequencing

Cluster generation was performed after applying 4 pM of each sample to individual lanes of an Illumina 1G flowcell. After hybridization of the sequencing primer to the single-stranded products, 18 cycles of base incorporation were carried out on the 1G analyzer according to the manufacturer's instructions. Image analysis and base calling were performed using the Illumina Pipeline and sequence tags were obtained after purity filtering. This was followed by sorting and counting of the unique tags. DGE libraries were registered in Gene Expression Omnibus (GEO, NCBI) under account number GSM537747 for uninfected salivary glands and GSM537746 for DENV-infected salivary glands.

### DGE tag annotation and comparison of expression data

Generation of expression matrices, data annotation, filtering and processing were performed using BIOTAG software (Skuld-Tech, France) [20]. First, flat files were downloaded from the GenBank (UniGene Build #12) and Ensembl (AaegL1, Jun 2009) databases. A table was constructed by extracting virtual tags from the representative sequences associated with each UniGene and Ensembl cluster file. Then BIOTAG functions were used for tag-to-gene assignment and subsequent data management. A query using tag sequence as the primary key allowed us to match experimentally obtained DGE sequences and virtual sequences with pre-selected annotations. Results are displayed in a table that provides the sequence of each DGE tag, its number of occurrences with the matching cluster number, its location in the sequence and other data extracted from the source file, including GenBank/Ensembl accession numbers.

The statistical value of DGE data comparisons, as a function of tag counts, was calculated by assuming that each tag has an equal chance of being detected. For several highly expressed transcripts, we checked that tag frequencies in successive sequence batches were distributed in agreement with a binomial law [20]. Selected genes were chosen based on a comparison between the two libraries, combined with the significance threshold of the observed variations (*p-value*<0.01).

### DENV-2 quantitative real-time PCR

Total RNA was extracted from salivary glands using RNable (Eurobio, France) following the manufacturer's protocol. The RNA was resuspended in 30 µL of RNAse-free distilled water and stored at −80°C. Subsequently, 0.6 µg of each RNA was reverse-transcribed using the SuperSript VILO cDNA Synthesis Kit (Invitrogen, Cergy Pontoise, France) following the manufacturer's instructions. TaqMan universal PCR master mix (Applied Biosystems, Courtaboeuf, France) was used in all qPCR procedures. Each reaction of 50 µl contained 300 nM of forward primer Denv_F 5′AGGACYAGAGGTTAGAGGAGA3′), 300 nM of reverse primer (Denv_R 5′CGYTCTGTGCCTGGAWTGAT3′), 150 nM of specific probe (Denv_P 6FAM_5′ACAGCATATTGACGCTGGGARAGACC3′_TAMRA) and 1× TaqMan universal PCR master mix. Primers and probe sequences targeted all dengue serotypes [21]. Amplification in an Applied Biosystem 7300 real-time PCR system involved activation at 95°C for 10min followed by 40 amplification cycles of 95°C for 15 sec, 60°C for 15 sec and 72°C for 30 sec. Real-time data were analyzed using SDS software from Applied Biosystems. Viral RNA was quantified by comparing the sample's threshold cycle (Ct) values with a the Dengue virus RNA standard curve which was obtained as follows: firstly, total viral RNA from the cell culture was purified using QIAamp Viral RNA kit (Qiagen, Courtaboeuf, France) following the manufacturer's protocol. Then, standard RT-PCR was carried out by using a primer containing the T7 promoter sequence (T7_Denv_F 5′TAATACGACTCACTATAGGAGGACYAGAGGTTAGAGGAGA3′, Denv_R 5′CGYTCTGTGCCTGGAWTGAT3′). The PCR product, containing the T7 promoter sequence was used to generate Dengue RNA fragments by *in vitro* transcription using the MAXIscript kit (Ambion, Austin Texas, USA). Then, RNA was purified by precipitation in sodium acetate and absolute ethanol. The amount of RNA generated was determined by spectrophotometry and converted to molecular copies using the following formula:
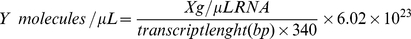
RNA standards containing 10^10^ to 10^2^ RNA copies were used to construct a standard curve.

### DENV immunolabeling

At various days following infection of female *Ae. aegypti* mosquitoes, DENV-infected and uninfected salivary glands were dissected in PBS and fixed in 100% acetone at −20°C for 1h. The tissue was incubated for 30 min in 10% goat serum 0.3% Triton X-100 to prevent non-specific staining and incubated overnight with the monoclonal 3H5 antibody which is directed against the DENV-2 envelope protein. Cells were washed with PBS and incubated overnight with phalloidin-Tetramethyl Rhodamine Iso-Thiocyanate (TRITC) at 4°C. Hoechst dye was used to stain the nucleus and preparations were examined with a confocal microscope Zeiss 5 Live Duo, as previously described [22].

### AAEL 000598 immunohistochemistry

5 days after the infection of female *Ae. aegypti* mosquitoes, DENV-infected and uninfected salivary glands and fat body were dissected in PBS and fixed with 4% paraformaldehyd at −20°C for 1h. The tissue was incubated for 30 min in 10% goat serum 0.3% Triton X-100 to prevent non-specific staining and incubated overnight with the AAEL000598-specific polyclonal antibody. Cells were washed with PBS and incubated overnight with Hoechst dye at 4°C to stain the nucleus. Preparations were examined as described above.

### Real-time PCR analysis of AAEL000598

cDNA was synthesized using 700ng salivary gland RNA (ten pooled salivary glands for each time point) or carcass and the MMLV reverse transcription Kit, following the manufacturer's protocol (Invitrogen, France). PCR reactions were run following the Roche Light Cycler LC480 protocol. PCR was performed using 1 µl of first cDNA, 3.33 µM of each primer and 2.5 µl of SYBR Green (Roche, France) in a 4 µl reaction volume. The cycling conditions were 40 cycles of 95°C for 10s, 57°C for 20s, and 72°C for 25s. RNA was quantified by calculating 2^−ΔΔCT^. Normalization was performed using a set of two internal control genes, the 40S ribosomal protein S17 and the ribosomal protein L28. The following specific primers were used: AAEL000598: forward 5′-GCTGTTCGCAATTGTGCTGTT-3′, reverse 5′-CAATTTCTTTCCCAGCTTCTTCA-3′; 40S ribosomal protein S17: forward 5′-CGCTGGTTTCGTGACACATC-3′, reverse 5′-TCTCTGCGCTCACGTTCCT-3′ and ribosomal protein L28: forward 5′-CCACGGTTAAGGTTACGCTGAA-3′, reverse 5′-CGACGGTAACGGTTCTTGTTG-3′.

### SELDI-TOF-MS

Surface-enhanced laser desorption ionisation time of flight mass spectrophotometry (SELDI-TOF-MS) Protein-Chip arrays (Biorad, France) were used as previously described [23]. Briefly, salivary gland extracts (from 4 groups of infected and uninfected mosquitoes) were diluted in a binding buffer (100 mM ammonium acetate, pH 4) and applied to the cation exchanger (CM10) chip. After 24h incubation at 4°C, unbound proteins were removed by three successive 5-minute washes with a buffer containing 100 mM ammonium acetate, Triton X-100 and 5 mM Hepes (pH 7). Chip-captured proteins were air-dried and covered with a matrix (3,5-dimethoxy-4-hydroxycinnapynic acid (SPA) in 99.9% acetonitrile and 0.1% trifluoroacetic acid), used to absorb laser energy. The ionized and desorbed proteins were detected and their molecular masses displayed on the proteogram. Peaks were determined using SELDI-TOF-MS analysis with Protein-Chip Biology System 3.5 software.

### Depletion of the 3.8 kDa MW protein from infected salivary glands

Depletion of the 3.8 kDa MW peptide from infected salivary glands was achieved by adding magnetic Bio-Adembeads coupled with protein G (Ademtech, France), pre-incubated for 2 h at 4°C with an anti-GK pAb After overnight incubation at 4°C followed by magnetic bead removal, the supernatant was analyzed by SELDI-TOF-MS.

The GK-specific polyclonal antibody was raised in rabbits following the immunization with the GK peptide and purified from immune serum by a GK-Sepharose affinity chromatography. This antibody binds to both uncleaved and cleaved AMP (data not shown).

### Peptide synthesis

*Ae. aegypti* peptides were chemically synthesized by Proteogenix (France) and checked by mass spectrometry (purity over 95%). The sequences of GK and MK peptide are: GK: GGLKKLGKKLEGAGKRVFKASEKALPVVVGIKAIGK; MK: MNMNFTKLFAIVLLAA LVLLGQTEAGGLKKLGKKLEGAGKRVFKASEKALPVVVGIKAIGK. The *Ae. aegypti* peptide (MT peptide) that has an irrelevant sequence, but a MW (6624.73 g/mol) similar to that of the MK peptide has been used as negative control. The sequence of the MT (AAEL000160) peptide is: MTLERIQETPALKGAPLSPLLRSLSGTLCMISQQRSVSHRT SKYSSNRHRKLQPFRET. In addition, a peptide (SC) of identical amino acid composition as the GK peptide, but with a scrambled sequence, has been used in the antibacterial assay. The sequence of the SC peptide is: VAKGLIKGVKAKGELPAKGVFKGLKESIGKRAVLKG.

### Antibacterial assays

*Escherichia coli* bacteria were cultured in LB medium with or without peptide. After overnight incubation at 37°C, bacterial growth was estimated by measuring the change in OD_600nm_ on a microplate spectrophotometer. The synthetic peptides' minimum inhibitory concentrations (MICs) of each of the synthetic peptides were expressed as the lowest concentration of the peptide that completely inhibited bacterial growth.

### Effect of peptides on DENV infection

The effect of peptides on viral production was assessed in permissive *Ae. albopictus* C6/36 cells infected with Dengue virus serotype 1–4 at a multiplicity of infection (MOI) of 1. Briefly, cells were exposed to DENV for 1 h at 4°C. Unbound virus was removed by washing cells 3 times with cold medium. Cells were then incubated for 24 h with different concentrations of GK or MK peptides (5 µg/ml and 10 µg/ml). The MT peptide at 10 µg/ml was used as a control in this experiment. Level of infection was determined by quantification of viral RNA by real-time PCR as described above.

### Measurement of viral titer by plaque assays

The consequence of peptide treatment on productive infection of mosquito cells was assayed as follows. Supernatants from DENV-infected C6/36 cells maintained in the presence of 10 µg/ml of peptides (GK, MK and MT) were collected at 24 h post infection (hpi). The production of viral particles in supernatants was determined by plaque titration assay on adherent permissive LLC-MK2 cells, which are permissive to all DENV serotypes. Briefly, LLC-MK2 cells were seeded in 6-well plates for 24 h at 37°C. After cell propagation, growth medium was removed and serial dilutions of Viral supernatants in DMEM 10% FCS were added to the cells. The inoculated cells were further incubated for 1 h at 37°C. Fresh 2× nutrient medium (Earle's balance salts supplemented with 0.07% (w/v) yeast extract, 0.33% (w/v) lactalbumin hydrolysate, 4% FBS, 7.5% NaHCO_3_, 2% essential amino acid) containing 1.5% agarose (Seakem) was added into each well volume by volume. The plates were incubated at 37°C, and plaques were visualized 5 to 7 days later, after addition of a second overlay of the above agarose solution supplemented with neutral red (0.1 mg/ml). The virus titre is expressed as Plaque Forming Unit (PFU) per millilitre.

### Effect of peptides on Chikungunya virus infection

Permissive HEK-293T cells were incubated with CHIKV (Strain 147-2-GFP) at a MOI of 1 and increasing concentrations of GK, MK or MT peptides. After 2 h at 37°C, viral input was removed and replaced by fresh medium supplemented or not with the peptides. After an additional 48 h in culture, the percentage of infected cells was directly related to the number of GFP-labelled cells counted by flow cytometry. A minimum of 10,000 cells were analyzed for each data point.

### Cytotoxicity assays

Cell viability was determined using a 3-(4,5-Dimethylthiazol-2-yl)-2,5-diphenyl tetrazolium bromide (MTT)-based assay (Sigma, France) [24]. Cells were set up and incubated with peptides under conditions that were identical to those used for antiviral assays. The cellular viability was determined measuring the absorbance values at 570 nm that were plotted against peptide concentrations to determine the IC_50_ values.

### Leishmanicidal activity

*Leishmania infantum* (strain MHOM/MA/67/ITMAP-263) and *Leishmania braziliensis* (strain MHOM/BR/75M2904) promastigotes expressing the luciferase gene were used to test the anti-leishmanial activities of the AMP as previously described [25], [26]. Briefly, 10^5^ promastigotes were inoculated into aliquots of 100 µl of medium in 96 well plates. After 4 h, the GK, MK or MT peptides were added and the plate was incubated for another 72 h before luciferase activity was assayed. Results are expressed as the relative light units (RLU) index ( = [RLU in treated wells/RLU in untreated wells]×100), whereas 100% represents parasite growth in the absence of peptides.

### Circular Dichroism (CD)

CD was performed to determine the peptides' secondary structures. CD spectra of peptide solutions in PBS or in different concentrations of trifluoroethanol (10%, 20% and 40%) were obtained using a Chirascan Circular Dichroism Spectrometer (Applied Photophysics, United Kingdom) at 20°C, with a water-jacketed quartz cell of 1 mm path length. Wavelengths from 188 to 260 nm were measured with a step resolution of 0.5 nm and a bandwidth of 5 nm. CD spectra were generated from an average of 2 scans of each sample. Secondary structures were determined using Chirascan software. The mean residue ellipticity [θ] (in degrees.cm^2^.dmol^−1^) was calculated using [θ] = [θ]_obs_(MRW/10.l.c), where[θ]_obs_ is the ellipticity measured in millidegrees, MRW is the peptide's mean residue MW, c is the concentration of sample in mg mL^−1^, and l is the optical path length in cm. Spectra were plotted as molar ellipticity [θ] versus wavelength.

### Accession numbers

The Ensembl or Entrez gene ID for the genes and proteins cited in the text is: AAEL000598 (putative antibacterial peptide, cecropin); AAEL000160 (hypothetical protein); AAEL014640 (peptidoglycan recognition protein-lc isoform); AAEL007064 (gram-negative bacteria binding protein); AAEL007619 (Toll); AAEL007768 (MYD88); AAEL003849 (hypothetical protein, defensin); AAEL003857 (hypothetical protein, defensin); AAEL009670 (lysozyme P); AAEL015639 (transferrin); 5565542 (diptericin).

## Results

### High-throughput deep sequencing of the Ae. aegypti salivary gland transcriptome response to DENV infection

**Figure 1:**
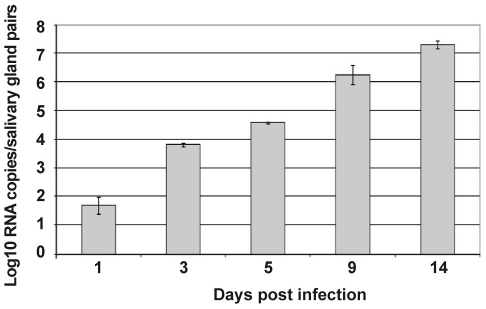
Follow-up of DENV-2 replication in the salivary glands from *Ae. aegypti*. At different time points after an infectious blood meal, propagation of DENV to salivary glands was investigated by real-time RT-PCR amplification of viral RNA in 80 pooled salivary glands for each time point post infection. Data are representative of three independent experiments (error bars represent standard error of the mean).

Salivary glands from *Ae. aegypti* females were collected at various time points following oral infection with DENV-2, and the kinetics of infection, as detected by the presence of viral transcripts, were monitored by real-time PCR. Viral transcripts could already be detected in the salivary glands 24 h after infection, albeit at very low levels (1.5 log10 RNA copies per pair of salivary glands). The expression of DENV transcripts increased over time until 14 days after infection (Figure 1). Because of the likelihood that salivary gland genes targeted by DENV are modulated with differential kinetics, we compared the transcriptomes of DENV-infected and uninfected salivary glands at a series of time points between 1 and 14 days after the blood meal, with the aim to follow the changes in the overall gene expression profile throughout DENV infection.

From this comprehensive analysis of gene expression profiles, more than 11 million DGE tags were sequenced. Among them, 39,912 unique tags with an occurrence of over 10 were detected. Thirty nine percent of these matched annotated genes in the GenBank and Ensembl databases (Supporting Information, Table S1). Based on criteria of probability (p<0.01) and the strength of induction (>2-fold), 1,111 transcripts were selected the expression of which was up-regulated in uninfected salivary glands, and 649 that were up-regulated in DENV-infected salivary glands (Supporting Information, Table S1).

### Salivary glands from DENV-infected mosquitoes overexpress a 3.8 kDa peptide

**Figure 2:**
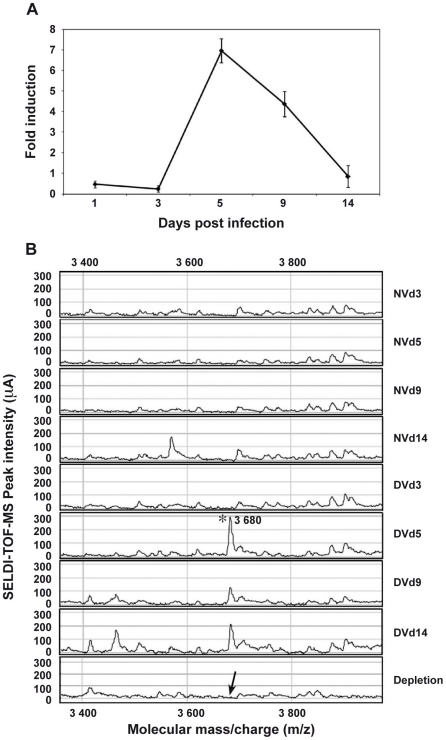
AAEL000598 mRNA expression and detection of the 3.680-kDa peptide in infected salivary glands. (A) Salivary glands were dissected from *Ae. aegypti* at 3, 5, 9 and 14 days after a blood meal containing or devoid of DENV, pooled for each time point (n = 10) and changes in the expression of AAEL000598 transcripts were measured using real-time PCR. Results are expressed as fold induction of AAEL000598 transcripts in DENV-infected salivary glands relative to those in uninfected salivary glands. (B) Salivary gland extracts from uninfected (NV) and DENV-infected (DV) mosquitoes were analyzed for the presence of the 3.680-kDa peptide by SELDI-TOF-MS. For depletion experiments, DENV-infected salivary gland extracts were incubated with a polyclonal Ab specific for the 3.680-kDa peptide coupled to magnetic beads and after removal of the beads reanalyzed by SELDI-TOF-MS. Data are representative of three independent experiments (error bars represent standard error of the mean).

Among the most strongly up-regulated genes in DENV-infected *Ae. Aegypti* salivary glands, we identified a gene belonging to the cecropin family (AAEL000598) (Table 1), a family of small cationic antimicrobial peptide that are induced in insects in response to hypodermic injury or bacterial infection [5]. Real-time quantitative PCR analysis was carried out to confirm the result obtained by DGE analysis and to investigate the chronology of the expression of this AMP in salivary glands following DENV infection. When compared with the expression profile observed in uninfected samples, a mRNA peak expression was detected 5 days post infection (dpi) in infected salivary glands (Figure 2A). Salivary glands were harvested from infected and uninfected mosquitoes at different time points to corroborate the over-expression of the AAEL000598 AMP, using the SELDI-TOF-MS technique. Results from this analysis revealed the presence of a peptide with a MW of 3.68 kDa that was more highly expressed in infected than in uninfected organs at 5, 9 and 14 dpi (Figure 2B).

**Table 1:** List of differentially expressed genes that are discussed in the text.

Tag sequence	lg	Nlg	P-value	Ensembl gene ID	Description	Chromosome
CATGAAGTGTTCTGAAAATAA	339	58	8.0E-57	AAEL000598	antibacterial peptide, putative	supercont1.12
CATGTCGAATAGCGAGCTCAA	9	1	5.7E-03	AAEL014640	peptidoglycan recognition protein-lc isoform	supercont1.1200
CATGAATGTTGCAGTCGGTGG	12	4	1.6E-02	AAEL007064	gram-negative bacteria binding protein	supercont1.233
CATGACGCAGATCGCGTTCAC	11	5	4.3E-02	AAEL007619	toll	supercont1.267
CATGGAGGAGAGAAAATTGAA	9	4	6.1E-02	AAEL007768	myd88	supercont1.279
CATGCAATATGAAAGATCTTT	790	323	6.6E-30	AAEL009670	lysozyme P, putative	supercont1.417
CATGAAAGGCAAGGGCAGTTG	24	10	2.9E-03	AAEL015639	transferrin	supercont1.4036
CATGAGTATTATTAGGATTGT	9	393	1.5E-92	AAEL003849	conserved hypothetical protein, defensin	supercont1.98
CATGCAGCCCCTCACTGTCAT	30	78	2.5E-05	AAEL003857	conserved hypothetical protein, defensin	supercont1.98

The table contains annotated DGE tags with specific occurrences generated for infected salivary glands (Ig) and uninfected salivary glands (NIg) libraries, *p-value*, the GenBank accession number and description, and the chromosome localization are listed to identify tags. Full lists are in the Web page (http://www.skuldtech.com/dengue_misse/).

**Figure 3:**
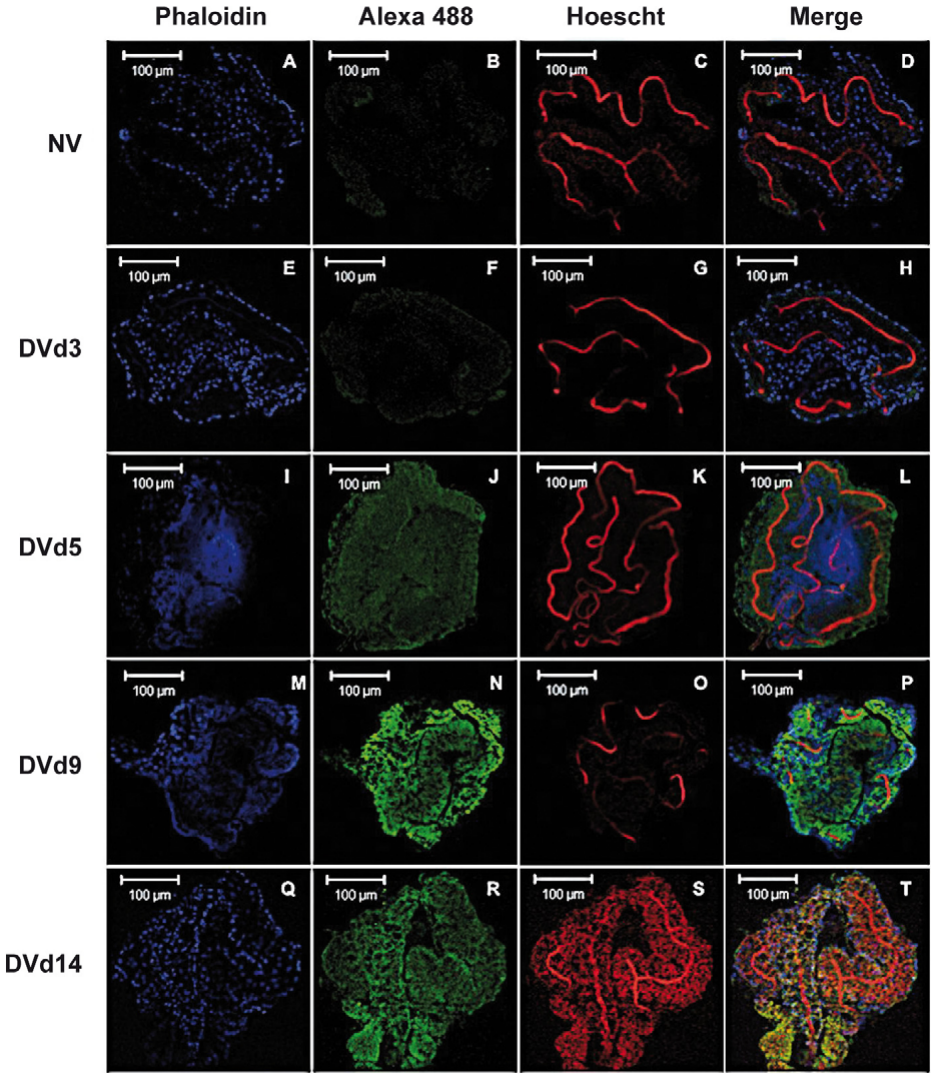
Immunofluorescence staining of DENV-2 envelope in infected *Ae. aegypti* salivary glands. (A–D) Uninfected salivary gland and DENV- infected salivary gland at (E–H) 3 dpi, (I–L) 5 dpi, (M–P) 9 dpi and (Q–T) 14 dpi. Nuclei are colored blue, DENV green and actin red. Data are representative of two independent experiments.

Incubation of 10 pooled salivary glands, collected at 5 dpi, with a polyclonal antibody raised against a synthetic peptide derived from the AEEL000598 sequence, immobilized onto protein G beads, followed by depletion of the Ab-peptide complex, resulted in the complete removal of the 3.68 kDa peak from the mass spectrometry peptide profile. This result confirms that the identified AMP in DENV-infected salivary glands corresponds to the cecropin AAEL000598 gene product (Figure 2B). Expression of AAEL000598 in salivary glands at 5 dpi was confirmed using immunofluorescence analysis (Supporting Information, Figure S1). In contrast, the peptide was not detected in the fat body at the same time point following DENV infection. Then we investigated whether the expression profile for AAEL000598 matched the kinetics of virus replication. Salivary glands isolated from mosquitoes at different time points post infection were prepared for confocal microscopy and labelled with a mouse monoclonal antibody specific for the DENV-2 envelope. Interestingly, the viral envelope protein was detected starting at 5 dpi (Figure 3). Our results suggest that the immune response against DENV in the salivary gland is activated early through intracellular signalling pathways that induce expression of this AMP.

### Antibacterial activities of the AMP

**Figure 4:**
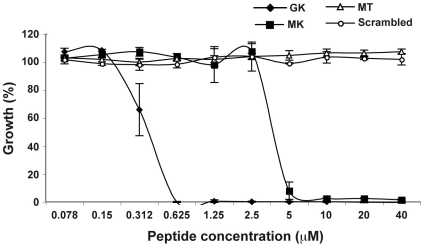
Antibacterial activity of GK and MK. *E.coli* were cultured in the presence of various concentrations of GK (diamond), MK (squares), MT (triangles) or SC (circles) peptides and bacterial growth was evaluated after 24 h of culture. Bacterial culture in LB medium only represents 100% growth. MT and SC peptides are used as negative controls. Data are representative of three independent experiments (mean ± SD).

In order to corroborate the supposed antibacterial activity of the identified AMP, its immature, signal sequence-containing, form (called MK) and its mature form (called GK) (Supporting Information, Figure S2) were chemically synthesized and their potency was evaluated in antibacterial tests. Both peptides were tested on the Gram-negative strain *E. coli* (Figure 4). Both the GK and the MK peptides killed *E. coli* in a dose-dependent manner with minimum inhibitory concentrations of 0.625 µM (GK) and 5 µM (MK), (Figure 4), respectively, which is consistent with a previous report showing that cecropins preferentially target gram-negative bacteria [27]. The *Ae. aegypti* MT peptide (AAEL000160) which has been reported in the Ensembl data base as a hypothetical protein with a MW similar that of MK peptide, used as a negative control in these experiments had no effect on bacterial growth. Similar results were also obtained with a peptide of identical amino acid composition to the GK peptide but with a scrambled sequence (Figure 4).

### Antiviral properties of the putative antimicrobial peptide

**Figure 5:**
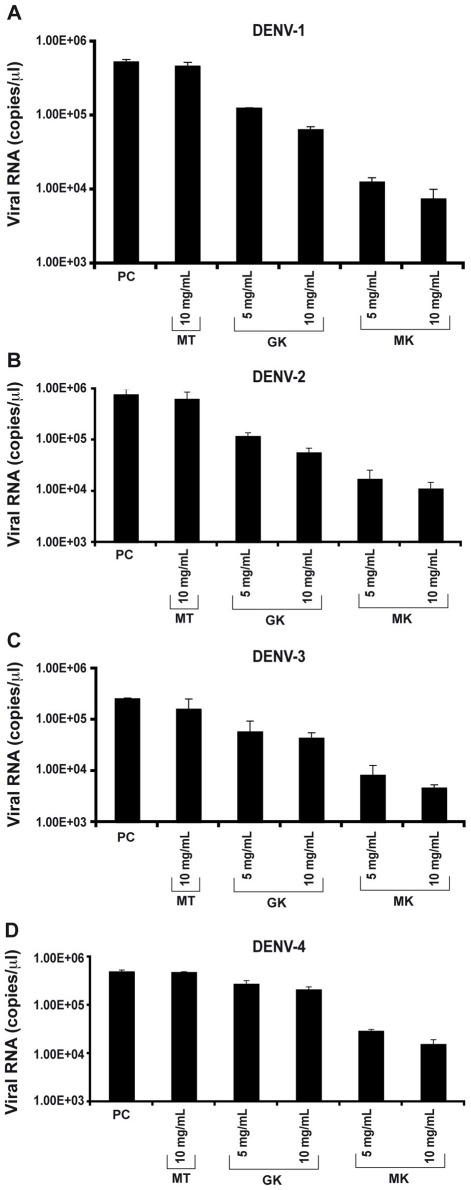
Inhibition of *in vitro* DENV replication by GK and MK peptides. C6/36 cells were incubated 1 h at 4°C with (A) DENV-1, (B) DENV-2, (C) DENV-3, (D) DENV-4 and then treated with 5 and 10 µg/ml of GK and MK peptides. DENV replication was analyzed after 24 h by real-time RT-PCR. Cells infected with DENV and maintained in the presence of medium alone (PC) or cultured with the MT peptide at a concentration of 10 µg/ml (MT) are used as controls. Data are representative of two independent experiments (mean ± SD).

Next, antiviral activity of GK and MK peptides against DENV replication was tested in permissive C6/36 cells infected with each of the four DENV serotypes and treated with various concentrations of the GK, MK or with the MT control peptide. Twenty-four h post-infection, quantitative real-time PCR was performed to measure viral RNA accumulation in C6/36 cells (Figure 5). The results show that intracellular viral RNA levels in DENV-infected C6/36 cells incubated in the presence of GK or MK peptides were reduced, in a dose dependent manner, when compared with levels detected from control cells infected with DENV and maintained in the presence of medium alone or cultured with the MT peptide. Under these experimental conditions, the MK peptide was approximately 10-fold more potent than the GK peptide, used at a similar concentration, and reduced the viral RNA by more than 1.5 log compared to the positive control. Similar results were obtained with all four DENV serotypes (Figure 5). From the two concentrations used, a more efficient inhibition was observed at a concentration of 10 µg/ml of either peptide. Neither peptide, at any of the concentrations used, generated cytotoxic effects as demonstrated by the IC_50_ values that were >290 µg/ml and 250 µg/ml for the GK and MK peptides, respectively.

The plaque reduction infectivity assay was also performed in virus-containing supernatants to determine virus count reduction as a result of treatment of infected C6/36 cells with the three peptides (Table 2). The amount of virus plaques was markedly decreased by about a hundred fold in the presence of the MK peptide and ten fold with GK peptide, as compared to infected cells, either untreated or treated with MT peptide. Furthermore, mean infectious titer correlated with RNA copy number, as determined by quantitative RT-PCR (Figure 5).

**Table 2:** Effects of GK and MK peptides on DENV growth.

	Plaque Forming Units/ml			
DENV serotypes	PC	MT	GK	MK
DENV-1 (Hawaii)	2.36×10^4^	2.03×10^4^	3.86×10^3^	3.80×10^2^
DENV-2 (16681)	3.50×10^4^	2.94×10^4^	2.57×10^3^	5.26×10^2^
DENV-3 (H87)	1.19×10^4^	9.74×10^3^	2.31×10^3^	2.18×10^2^
DENV-4 (814669)	2.54×10^4^	2.01×10^4^	1.33×10^3^	6.78×10^2^

C6/36 cells were infected with DENV (serotypes 1–4) at a MOI of 1 and were cultured in the absence (PC) or the presence of 10 µg/ml of each of the GK, MK or the control MT peptides. After 24 h of culture, supernatants were collected and the presence of DENV was determined by plaque assay. Plaque Forming Units/ml in cell culture supernatant are presented as the mean of four replicates from two independent experiments.

**Figure 6:**
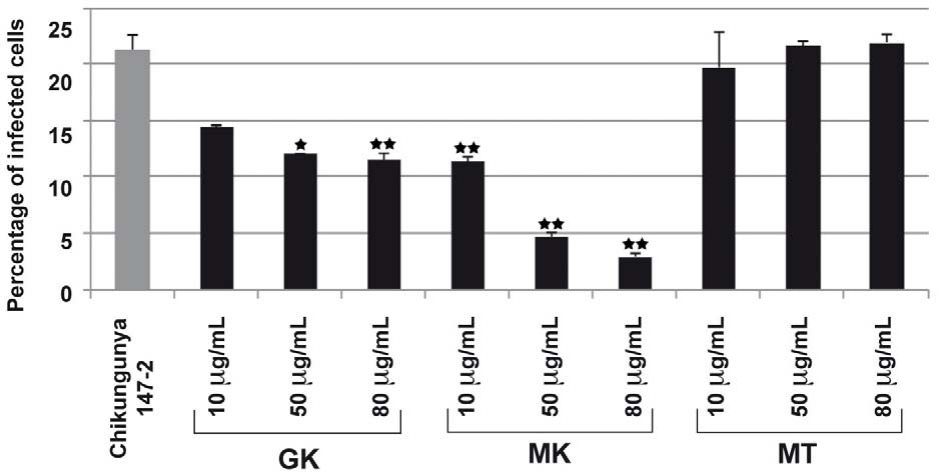
GK and MK CHIKV antiviral activity. 293T cells were infected with the CHIKV 37997 strain used at a MOI of 1 for 1 h at 37°C in the presence of various concentrations of GK, MK or MT peptides. After 12 h in culture, the percentage of CHIKV-infected cells was monitored by the detection of intracellular CHIKV antigens using a polyclonal anti-CHIK serum and FITC-conjugated secondary antibodies. Expression levels were quantified by flow cytometry. MT peptide is shown as control. Each value is the mean of 2 separate experiments performed in duplicate (mean ± SD). One-way ANNOVA was employed to analyze the differences between sets of data. A value of p<0.05 was considered significant. * indicate p values<0.05 and ** p values<0.01.

To further investigate the antimicrobial properties of the cecropin-like peptides, we evaluated their inhibitory effects in a CHIKV model of infection. To this end, we used a model of HEK293T cells. This human epithelial cell line, was reported to be susceptible to CHIKV infection [28], [29] and was thus used as an indicator cell line for viral infectivity. As shown in Figure 6, both GK and MK peptides inhibited the infection of HEK-293T cells by the CHIKV 147-2 strain in a dose dependent way. The MK peptide was more potent with inhibition levels of about 50% at 10 µg/ml and 80% at 80 µg/ml, respectively. Under these experimental conditions, the control MT peptide induced no reduction of cell infection at any of the concentrations used. It is important to note that the GK IC_50_ was >290 µg/ml and that of the MK peptide was 200 µg/ml, indicating that none of the peptides had cytotoxic effects used at these concentrations.

### Leishmanicidal activity

**Figure 7:**
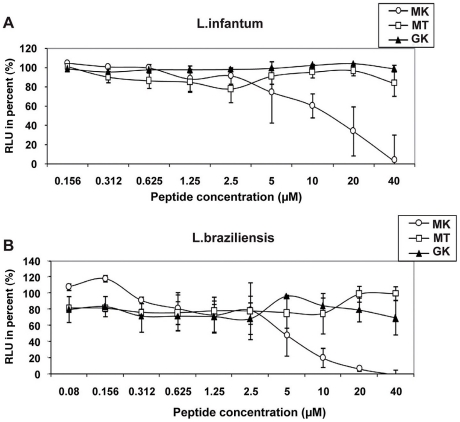
Leishmanicidal activity of GK and MK. The effect of MK (circles), MT (squares), and GK (triangles) peptides on the luciferase gene-containing promastigotes (A) *L. infantum* and (B) *L. braziliensis* was determined after 72 h of exposure of the parasites to various concentrations of the peptides. Untreated cultures correspond to parasite growing in culture medium only. The RLU percentage was calculated for each test sample according to the following formula [RLU in treated wells/RLU in untreated cultures]×100. Results represent mean values ± SD of three independent experiments.

Finally, in order to determine the spectrum of anti-pathogenic activity of the GK and MK peptides, their capacity to kill the human protozoan parasites *L. infantum* and *L. braziliensis* promastigotes, was evaluated as well (Figure 7). Of note, whereas the MK peptide mediated potent activity against both parasites with respective IC_50_ values of ∼15 µM (Figure 7A) and ∼10 µM (Figure 7B), the GK and the MT control peptides were inactive.

### Circular dichroism

**Figure 8:**
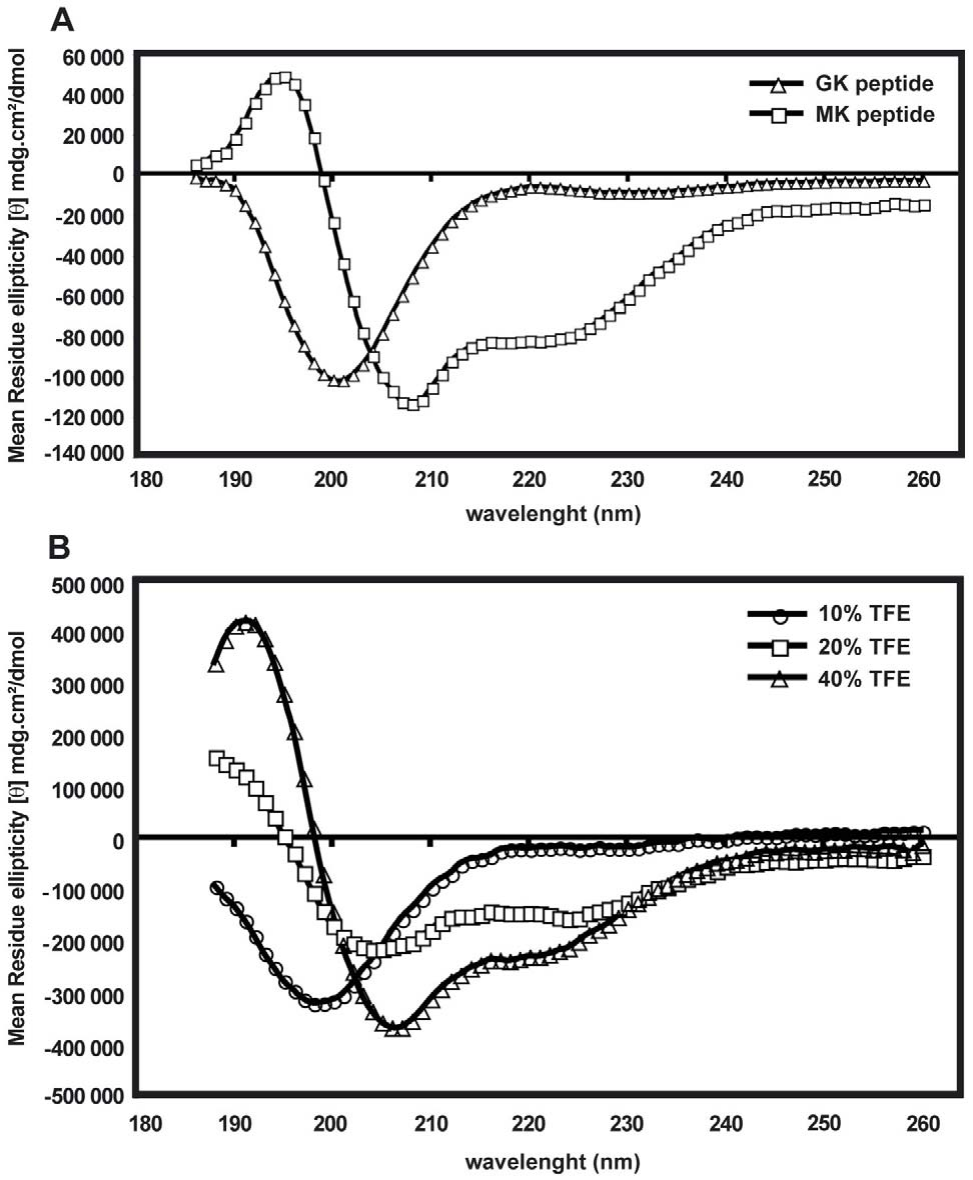
Folding of GK and MK peptides in circular dichroism experiments. (A) CD spectra of the GK peptide (triangles) and the MK peptide (squares) in PBS. (B) CD spectra of the GK peptide in 10% TFE (circles), 20% TFE (squares) and 40% TFE (triangles). Data are representative of two independent experiments.

The difference between the GK and MK peptides in their ability to display antimicrobial activity might be due to differences in their molecular conformation (Supporting Information, Figure S2). Therefore, circular dichroism (CD) spectra were used to study the content of secondary structures of both peptides. Figure 8A represents the CD spectra of the GK and MK peptides at 25°C in PBS and shows that the MK peptide contains α-helical structures, as indicated by a negative ellipticity at wavelengths of 208 and 222 nm, as well as a CD peak at 195 nm. In contrast, the CD spectrum of the GK peptide shows a negative band around 200 nm which is typical for the back bond of a peptide in random-coil conformation. In order to test whether the GK peptide was able to adopt a helical structure in a hydrophobic environment, it was diluted in PBS containing various proportions of TFE (10–40%) (Figure 8B). With an increasing concentration of TFE, the conformation of the GK peptide gradually changed from a random-coil (10% TFE/PBS) to an α-helix structure in a relatively hydrophobic environment (40% TFE/PBS). This property is shared by many membrane-binding peptides [30]. In contrast, the MK peptide maintained its helical conformation independently of the concentration of TFE (data not shown).

### Other immune-related genes regulated during DENV infection of salivary glands

Another strongly up-regulated gene, with a 9-fold over-expression in DENV-infected salivary glands, was identified as the membrane-spanning peptidoglycan recognition protein (PGRP-LC, AAEL014640) (Table 1). The PGRP-LC protein is known to be important in innate immune responses in *Drosophila* [31].

A member of the Gram-negative bacteria-binding protein (GNBP) family (AAEL007064), the Toll-like receptor Toll5A (AAEL007619) and the MYD88 factor (AAEL007768), were also up-regulated in infected salivary glands (Table 1), suggesting that the Toll pathway is activated in *Ae. aegypti* salivary gland anti-dengue defense mechanisms.

Interestingly, the most significantly down-regulated gene detected encodes a conserved hypothetical protein (AAEL003849) that is also an AMP belonging to the defensin family (Table 1). Moreover, the expression of another defensin gene (AAEL003857) was also found to be down-regulated in infected salivary glands. These results underscore the ability of the virus to survive the rigor of the mosquito's immune system.

Lysozyme P (AAEL009670) is another immune-related mosquito gene [32] with enhanced expression in DENV-infected salivary glands (Table 1). Lysozyme C which has been linked to melanization reactions has been shown to be up-regulated in the midgut following DENV infection of *Ae. aegypti* [8], thereby pointing to the importance of lysozyme family in the host defense mechanisms of this vector. In our study, transferrin (AAEL015639) was induced by DENV infection of the salivary gland (Table 1). It has been shown that transferrin synthesis and secretion are increased when *Aedes* mosquito cells are exposed to bacteria, suggesting that mosquito transferrin may act as an acute-phase protein [33].

## Discussion

DENV is not simply passively transported to humans by its vector *Ae. aegypti*; rather, it intimately interacts with the mosquito, allowing viral multiplication that induces significant biochemical and molecular changes in the host. Following the ingestion of an infectious blood meal, replication of DENV occurs in different mosquito tissues, including the salivary glands. Viral replication in this compartment is a prerequisite for subsequent injection of infectious saliva into the human host and continuation of the DENV transmission cycle. Accordingly, it is of crucial importance to elucidate the immune response mounted against DENV in this particular compartment for future elaboration of antiviral strategies.

The comprehensive transcriptome analysis of the *Ae. aegypti* salivary gland in response to DENV infection performed in this study has allowed us to identify unique sequences of genes the products of which are modulated following viral infection. It is of note that, despite the availability of the completely sequenced genome of *Ae. Aegypti* [10], a significant number of tags unmatched with annotated databases was observed. Unmatched tags might correspond to novel transcripts not yet identified in the *Aedes* genome, including alternatively spliced transcripts from known genes, as well as transcripts from novel genes. The latter possibility is underscored by the recent demonstration that the use of novel DGE tags as probes has permitted the identification of transcripts and genes in the human genome that were difficult to identify by conventional methods [34].

Among the modulated tags, the AAEL000598 sequence was the most strongly up-regulated gene in DENV-infected salivary glands. This sequence encodes a small cationic AMP belonging to the cecropin family [5]. The DENV-induced up-regulation of the AAEL000598 gene resulted in the production of a cecropin-like peptide at 5 dpi, the identity of which was confirmed using an antibody specific for the AEEL000598 gene product. Both the RNA and protein expression patterns concur with the detection of viral antigen early after the blood meal (5 dpi). This result corroborates a recent study suggesting that the extrinsic incubation period (EIP) for DENV may be shorter than previously reported and that it depends on the nature of the viral strain, as well as the genetic background of the vector [4]. As an example, short EIPs have been observed for other vector-arbovirus interactions: EIPs are only 2 days for CHIKV in *Ae. Aegypti* [35], for Rift Valley fever virus in *Culex pipiens* [36] and for Venuezuelan Equine Encephalitis virus in *Ae. aegypti* [37]. With respect to our data, the presence of DENV RNA, associated with an absence of envelope expression in salivary glands at 24 hpi, suggests either that the viral particles present in this tissue at this time point are incomplete, or that there is not yet enough virus present to allow detection of the viral envelope by confocal microscopy. Investigating the assembly of entire particles, for example by electron microscopy imaging, would help to discriminate between these hypotheses.

Fat body tissue has been reported to express cecropin as well and it is therefore important to rule out potential contamination of the salivary glands, used for the DGE analysis, by the surrounding fat body. The induction of cecropin in fat body tissue by DENV is however rapid and transient, as, following its expression 24 hpi, it is no longer detected at 3 dpi [38]. Therefore, the absence of cecropin expression in fat body tissue at 5 dpi, as demonstrated in the present study, argues for a lack of contamination during the dissection procedure. To further rule out contamination of the salivary gland samples by fat body tissue, we also focused on diptericin expression in both tissues. Tag-DGE analysis of the salivary glands RNA libraries revealed a maximum of 3 diptericin tag-DGE counts for 6 million tag-DGEs sequenced per library. This result is very close to background. Given that diptericin is highly expressed in fat body tissue [38], these results confirm the absence of contamination of samples used in the present study.

The functional properties of the AEEL00598-encoded cecropin-like peptide were determined using two synthesized peptides MK and GK, corresponding to the pre-protein and mature product, respectively. Both peptides were found to display broad anti-infectious properties and were active against DENV, CHIKV, as well as against *E. coli* bacterial strain. However, in some experiments the immature peptide was significantly more potent than the mature peptide used at a similar concentration: although CHIKV infection was less sensitive to the antiviral properties of the immature peptide, both peptides interfere with Flaviviruses and Alphaviruses, viral pathogens that belong to two different families of RNA viruses. Moreover, the immature peptide was efficacious against *Leishmania* while the mature was not, thus confirming that the immature form of the cecropin peptide has an even broader spectrum of activity, being able to inhibit pathogens vectored by *Aedes* mosquitoes, as well as a parasite like *Leishmania* that is not transmitted by mosquitoes. Based on the results from CD spectra analysis, these differential effects may be due to conformational differences, which are associated with the presence of the signal sequence in the immature form of the peptide. However, from a mechanistic point of view, the mode of action of AMPs, their selectivity for certain pathogens, as well as their lack of activity against normal eukaryotic cells, remain yet to be determined. This selectivity appears to depend on the lipid composition of the target membrane which determines its fluidity and charge the microbial membrane showing a highly negative electrical potential) and whether or not cholesterol is present. Indeed, the plasma membrane of the *Leishmania* promastigote shows significant differences from those of other eukaryotic cells [39], [40] because it has a high negative charge due to high levels of lipophosphoglycan and contains a higher proportion of anionic phospholipids. In this context, as evidenced for bacteria, AMPs are able to alter the structure of biological membranes as a result of the binding of positively charged regions of their α-helical peptides to negatively charged lipids in the membrane [41]. It has been suggested that AMPs above and beyond their ability to permeabilize and disrupt membranes may affect microbial viability by interacting with intracellular targets or disrupting key intracellular processes *via* the alteration of cytoplasmic membrane septum formation, the inhibition of cell-wall, nucleic-acid and protein synthesis, as well as the inhibition of enzymatic activity, following their translocation across the plasma membrane (review in [42]. Such properties could explain virucidal activity of α-helical peptides. In addition, it is possible that such peptides could inhibit viral replication by interfering with membranes of the endoplasmic reticulum system, as DV replication is known to depend on the integrity of these membranes [43]. Results from structural characterization by Nuclear Magnetic Resonance analysis and the determination of the crystal structure of the peptides will provide more insight into their mode of action.

Cecropin family peptides do have anti-pathogenic activities. A recent study showed that co-overexpression of two AMPs, cecropin A and defensin A, in transgenic *Ae. aegypti* mosquitoes results in a cooperative antibacterial and anti-plasmodium action [44]. Moreover, peptides of the cecropin family reportedly possess anti-HIV activity [45]. Finally, scorpine, a scorpion AMP that resembles a hybrid between a defensin and cecropin was shown to have antibacterial and antiplasmodial activity, as well as antiviral activity against DENV [46]. Such properties encourage the design of analogs consisting of sequences containing single amino acid replacements or hybrid peptide sequences derived from cecropins or cecropins with other AMPs, such as thanatin, melittin, magainin and temporin. In light of the antipathogenic properties observed for the MK and GK peptides, it is to be expected that in the near future a rational design approach based on these peptides may yield derived products that are more effective at lower concentrations. For example, the C-terminal amidation of these peptides might enhance their stability and cationicity, as has been demonstrated for the C-terminally amidated cecropin A of the giant silk moth *Hyalophora cecropia* which is more active than cecropin A itself [47]. Otherwise, as the antimicrobial activity of several of these analogs is more potent than that of their parent molecules [48], hybrid molecules based on the structure of MK and GK peptides could be powerful effectors. Insect AMPs have been selected throughout evolution for their low toxicity to eukaryotic cells. Over the past decade, strains of many common microbes have continued to develop resistance to drugs [49] and, because of the urgent need for novel treatment modalities, insect AMPs could serve as a template for the design of novel therapeutic compounds.

Besides the AAEL000598 gene, several other genes were identified the expression of which was up-regulated following DENV infection of *Ae. Aegypti* salivary glands. Among these, the PGRP-LC protein is known to be important in innate immune responses in *Drosophila* [31], being involved in the signal transduction events that lead to the induction of AMP gene expression. PGRP-LC recognizes molecular patterns in exogenous microbes and activates the IMD pathway in response to pathogen infection [50], [51]. This pattern recognition receptor could therefore be involved in the up-regulation of expression of the AAEL000598 gene.

Up-regulation of GNBP (AAEL007064), Toll5A (AAEL007619) and MYD88 (AAEL007768) gene expression in infected salivary glands supports the notion that the Toll pathway is activated following DENV infection of *Ae. Aegypti*. This result furthermore corroborates a recent study showing that silencing of MYD88, a key component of the Toll pathway, results in a small but significant increase in DENV load in the midgut of infected mosquitoes [8]. Accordingly, following infection the Toll pathway regulates expression of antiviral molecules. Interestingly, a mosquito RNAi-mediated silencing of *Cactus*, a negative regulator of the Toll immune pathway, has been shown to induce the expression of innate immune response-related genes including AAEL000598 [8]. Genetic analysis of the systemic immune response of *Drosophila* has indicated that activation of the Toll pathway accounts primarily for the response of this invertebrate to infections by fungi and Gram-positive bacteria [52], whereas the IMD pathway is mainly induced in response to Gram-negative bacterial infection. For example, in *Drosophila*, cecropin A is regulated preferentially, but not exclusively, by the IMD pathway [53]. Although both pathways can be activated independently, they usually function synergistically, as has demonstrated in flies infected with the *Drosophila* C virus [54], [55]. The interaction between both pathways during the infection of mosquitoes by DENV remains however to be established.

Like the midgut, the *Ae. aegypti* mosquito salivary gland compartment harbours a potent cellular response against DENV. The results from the analysis of the *Ae. Aegypti* salivary gland transcriptome reported in the present study show that many of the genes that are up-regulated following infection with DENV, encode proteins that are involved in the innate immune response and that participate in the IMD signalling pathway. Recent studies have provided important insights into *Ae. aegypti* immune responses to DENV-2 infection in the midgut [8], [9], [38] showing that the Toll and the JAK-STAT pathways, as well as the RNAi machinery are important for the mosquito's defense against DENV infection. No involvement of the IMD pathway in the control of DENV-2 was reported in these studies, but this is to be expected since the analyses were carried out 10 dpi, whereas elicitation of the IMD pathway represents an acute response [56]. Together with these data, the results presented here demonstrate that both the IMD and Toll-like signaling pathways are modulated following infection of the salivary gland by DENV-2. This is quite consistent with the very recent data in the literature, reporting up-regulation of cecropin in the midgut and fat body 24 h after oral infection of the mosquito with DENV-2 [38]. Studies on the survival of DENV-infected mosquitoes in which one of these signaling pathways is defective could shed light on the molecular mechanisms that underlie the interplay of these pathways, as well as on the regulation of their respective activity.

The effectiveness of defense of the vector is likely to rely on the arsenal of strategies present in the midgut, fat body and salivary glands, which help the insect survive in a hostile environment that is harboring a wide variety of potential pathogens. It is nevertheless important to stress that, in spite of these immune response mechanisms, DENV accumulates viral genome RNA and infectious virus in the midgut and salivary gland, which is subsequently transmitted to humans. This underscores the complexity of the interaction between the virus and its vector, as well as the notion that the escape mechanism of the virus within the vector remains poorly explored. From an evolutionary point of view, the mosquito immune response has evolved to fight the infection until its detrimental fitness consequences are eliminated, albeit not to fully eradicate the virus. In this context, it is not paradoxical that mosquitoes can both mount an apparently robust response to virus infection and remain competent vectors. Further studies would be necessary to test this hypothesis. It would also be interesting to carry out RNAi-mediated knockdown studies of this peptide to understand his contribution in the antiviral signalling pathways.

Finally, the results from this transcriptome analysis will facilitate the understanding of the molecular mechanisms of the insect-virus relationship and could help to develop novel strategies to reduce the transmission of viral diseases, including the use of transgenic insects or the identification of novel sources of pharmacological compounds.

## Supporting Information

Figure S1 Expression of cecropin in *Aedes aegypti* salivary gland and fat body. Infected salivary gland (A), non-infected salivary gland (B), infected fat body (C) and non-infected fat body (D) were dissected at day 5 post infection. Cecropin was visualized in green by immunohistochemistry assay. Nucleuses were stained in bleu by Hoechst.
(0.41 MB EPS)

Figure S2 Secondary structure prediction of MK and GK peptides. The predicted secondary structures for (A) MK and (B) GK were generated using the Advanced Protein Secondary Structure Prediction Server [57]. The lines in the order are the original amino acid sequence, string of the predicted structure and confidence level of the secondary structure prediction. The amino acid sequence corresponding to the signal peptide, the putative cleavage sites for signal peptidase (black arrow) and α-helix (H), β-sheet (E) coiled coil (C) structures are represented.
(0.49 MB EPS)

Table S1 List of differentially expressed genes. The Web page (http://www.skuldtech.com/dengue_misse/) contains filtered DGE data and is classified in 3 pages: annotated tags up- and down-regulated, and unknown tags. Each table includes the tag sequence (CATG+10nt), specific occurrences of each DGE tag, generated for infected salivary glands (Ig) and uninfected salivary glands (NIg) libraries, and *p-value*. The direct web link to Ensembl database, the GenBank accession number and description, and the chromosome localization are listed to identify tags.
(0.02 MB DOC)
