# Supplementary material for: Induction of a Peptide with Activity against a Broad Spectrum of Pathogens in the Aedes aegypti Salivary Gland, following Infection with Dengue Virus
Source: PLoS Pathog. 2011 Jan 13;7(1):e1001252. doi: 10.1371/journal.ppat.1001252 (PMC3020927; doi:10.1371/journal.ppat.1001252)
Supplement: Table S1 — List of differentially expressed genes. The Web page (http://www.skuldtech.com/dengue_misse/) contains filtered DGE data and is classified in 3 pages: annotated tags up- and down-regulated, and unknown tags. Each table includes the tag sequence (CATG+10nt), specific occurrences of each DGE tag, generated for infected salivary glands (Ig) and uninfected salivary glands (NIg) libraries, and p-value. The direct web link to Ensembl database, the GenBank accession number and description, and the chromosome localization are listed to identify tags. (0.02 MB DOC) [file ppat.1001252.s003.doc]

**Table S1.** **List of differentially expressed genes.**

The Web page (http://www.skuldtech.com/dengue_misse/) contains filtered DGE data and is classified in 3 pages: annotated tags up- and down-regulated, and unknown tags. Each table includes the tag sequence (CATG+10nt), specific occurrences of each DGE tag, generated for infected salivary glands (Ig) and uninfected salivary glands (NIg) libraries, and *p-value*. The direct web link to Ensembl database, the GenBank accession number and description, and the chromosome localization are listed to identify tags.
